# The Pros of Not Being Competitive

**DOI:** 10.2174/157015907781695964

**Published:** 2007-09

**Authors:** VINCENT MUTEL, BERNHARD BETTLER

Drugs acting at G-protein coupled receptors (GPCRs) represent the core of modern medicine. Traditionally, these drugs target the orthosteric site of the receptors, i.e. the binding site of the endogenous agonist. An increasing number of patents describe synthetic allosteric modulators of GPCRs, which influence receptor activity at sites distinct from the orthosteric site. This documents that the search for allosteric modulators of GPCRs has become a fully-fledged part of current drug discovery efforts. It is now generally accepted that allosteric modulation of GPCRs holds considerable promise for the development of novel therapeutics, but it has taken decades to persuade the skeptics of the advantages this concept has to offer. Allosteric modulation is now considered a valid strategy to obtain first generation therapeutics as well as second generation alternatives to currently available therapeutics. A key step towards broad exploration of the allosteric concept was the introduction of cell-based functional high-throughput screening assays for GPCRs, which have replaced radioligand binding assays as the primary screen for allosteric compounds. The reviews in this special issue, written by some of the leading scientists in the field, provide an overview of the experimental strategies used to identify and characterize allosteric modulators for Class A, B, and C GPCRs.

There are a number of theoretical and practical reasons that support the notion that it is easier to obtain selective and/or effective therapies with allosteric modulators than with conventional competitive ligands. Most importantly, allosteric modulation enables the development of molecular entities that have no equivalent in the world of competitive ligands. With a range of allosteric compounds it is, in principle, possible to stabilize receptors in various biologically active conformations, which allows the chemical adjustment of receptor activity in more sophisticated ways than with orthosteric ligands. It is possible not only to identify allosteric ligands that act as positive or negative allosteric modulators of GPCR function, but also to identify allosteric ligands that act as agonists in their own right. Positive and negative allosteric modulators can affect both affinity and efficacy of the endogenous agonist, thereby significantly expanding the pharmacological repertoire for a given target. Allosteric modulation may sometimes also be the most straightforward way to identify small molecular weight compounds for targets that are already pharmacologically or clinically validated. This is particularly evident for Class B GPCRs, which are activated by peptide ligands and normally display poor chemical tractability. Allosteric modulation therefore offers the opportunity to develop non-peptide therapeutics that can be administered orally and that readily cross the blood-brain barrier.

A primary goal in pharmaceutical research is to selectively target novel drugs to defined receptor populations, which is expected to reduce side effects and to expand the range of clinical applications. Given the generally high degree of sequence conservation within orthosteric binding sites of GPCRs, competitive ligands have been very disappointing in terms of their subtype-selectivity. It has been much easier to achieve subtype-selectivity with allosteric ligands that, for example, clearly distinguish members of the metabotropic glutamate receptor or muscarinic receptor families. This supports the concept that the binding sites for allosteric ligands are less conserved than the binding-sites for orthosteric ligands. Because the allosteric and orthosteric sites are conformationally linked, an allosteric modulator could essentially produce a receptor with completely novel reactivities towards the endogenous agonist. subtype-selective allosteric compounds may therefore additionally impose a degree of functional selectivity to the receptor that contributes to subtype-specific effects. Moreover, since binding of an allosteric ligand can change the interfaces between the receptor and effector molecules, it may be possible to selectively influence a subset of possible signaling pathways. Importantly, from a drug discovery point of view, it is becoming clear that allosteric ligands are less limited in pharmacophore diversity than orthosteric ligands. This increases the likelihood of solving e.g. toxicology or bioavailability problems along the drug discovery process. Pharmacophore diversity suggests the existence of topographically distinct allosteric binding sites within individual GPCRs. Supporting evidence for this comes from the analysis of allosteric binding-sites in metabotropic glutamate receptors. Allosteric sites at the interface of receptor subunits could therefore be used to potentially dissociate the effects of homo- and heteromeric GPCRs.

Positive allosteric modulators possess the advantage that they discriminate between activated and non-activated receptor states, while agonists indiscriminatingly activate all receptors. Allosteric modulators may therefore have a broader therapeutic window than agonists. This was directly shown to be the case for the allosteric modulators of GABA_B_ receptors, which retain the therapeutic properties of agonists in the absence of typical side-effects. In addition, positive allosteric modulators carry a reduced liability for receptor desensitization and/or tolerance, which can drastically expand the range of possible therapeutic applications. Partial negative allosteric modulators enable the adjustment of the activity of GPCRs towards a predefined level, without completely blocking activation of the receptor. This may be useful in circumstances where a receptor mediates pathological functions while at the same time mediating physiologically useful functions. First examples for a permissive antagonism are emerging, where it has been possible to block a pathological response while leaving normal physiological responses intact. 

In principle, allosteric modulation offers the opportunity to stabilize small molecular differences in receptor conformations and to adjust receptor activity in subtle ways. The challenge now is to identify the therapeutically most useful profiles for allosteric drugs. Unfortunately, an insufficient mechanistic understanding of GPCR signaling under normal and pathological conditions often hinders straightforward allosteric ligand optimization. Nevertheless, the available data on the effects of allosteric compounds *in vivo* suggest that many expectations regarding efficacy, selectivity and safety hold true. While research compounds illustrate the potential advantages of using allosteric compounds for therapy, the calcimimetic cinacalcet is the only allosteric modulator acting on a GPCR that has so far reached the market. As such, cinacalcet constitutes a most important proof-of-concept step for future development of allosteric modulators in the GPCR field.

## Statement of Conflict of Interest

Vincent Mutel is CEO and Bernhard Bettler a member of the advisory board of Addex Pharmaceuticals, Geneva

